# New approach to managing occult hepatitis B infection

**Published:** 2011-04-01

**Authors:** Murat Sayan

**Affiliations:** 1 Clinical Laboratory of Kocaeli University Hospital, Kocaeli, Turkey

**Keywords:** Hepatitis B virus, Hepatitis

Dear Editor,

I read with great interest the paper by Taheri, et al. published in [1] Hepatitis Monthly (Number 31, February 2011). The results of this follow-up study are hopeful of hepatitis-B vaccine efficacy in patients who lost HBsAg and did not seroconvert to anti-HBs with no detectable HBV DNA. However, the editorial in the same issue, as well as Taheri et al. themselves, noted some important limitations to this study [2]. According to the findings of a meta-analysis by Poorolajal et al. [3], the protection provided by the HBV vaccine is dependent on immune memory rather than circulating anti-HBs titer. Therefore, it would be worthwhile to observe the anamnestic immune response to HBsAg in such patients. Furthermore, such an investigation should include observations of spontaneous developing anamnestic immune response. The authors prefer an artus HBV RG PCR kit for HBV DNA detection but not for quantification. Because of the lower analytic sensitivity (10.22 IU/ml), the artus HBV QS-RGQ kit (QIAGEN GmbH, Hilden Germany) would be an incorrect chocie for this study design. Therefore, absent of measuring the HBV viral load as stated in the editorial, the HBV RG PCR kit does not present any limitations for following-up with occult hepatitis B patients.

In Taheri, et al.'s study, some patients with undetectable HBV DNA require more explanation. Specifically, they test negative for HBsAg but still test positive for HBeAg [Study Groups 2 and 3 (4%)]. However, seroconversion to anti-HBs in these patients is not clear. Also, the patients in Group 1, who tested negative for HBsAg but had detectable amounts of HBV DNA, should be analyzed for typical HBsAg escape mutation. The literature has discussed that some emergent mutations in the S gene region of HBV (naturally occuring or dependet to oral antiviral using) may cause diagnostic problems for some of the typical HBsAg escape mutations and may not be detected in HBsAg assays [4]. Patients who have or are infected with diagnostic escape HBsAg mutation cannot be considered as lost HBsAg. Finally, despite a low protection rate [5] of anti-HBs antibody level (68 ± 32.66) in a small number of patients, the findings of Taheri et al.'s study establishes that a new approach to managing occult hepatitis B infection may be to use nucleos(t)ide analogue treatments on patients who test negative for HBsAg.
